# The Role of Light in the Emergence of Weeds: Using *Camelina microcarpa* as an Example

**DOI:** 10.1371/journal.pone.0146079

**Published:** 2015-12-30

**Authors:** Aritz Royo-Esnal, Russell W. Gesch, Frank Forcella, Joel Torra, Jordi Recasens, Jevgenija Necajeva

**Affiliations:** 1 Dpt. D’Hortofruticultura, Botànica i Jardineria, Agrotecnio, Universitat de Lleida. Alcalde Rovira Roure 191, 25198, Lleida, Spain; 2 Dpt. Plant Physiology, University of Latvia. Kronvalda bulv. 4, 1586, Riga, Latvia; 3 USDA-ARS-North Central Soil Conservation Research Laboratory, 803 Iowa Avenue, Morris, Minnestoa, 56267, United States of America; California State University, Fresno, CA, UNITED STATES

## Abstract

When modelling the emergence of weeds, two main factors are considered that condition this process: temperature and soil moisture. Optimum temperature is necessary for metabolic processes that generate energy for growth, while turgor pressure is necessary for root and shoot elongation which eventually leads to seedling emergence from the soil. Most emergence models do not usually consider light as a residual factor, but it could have an important role as it can alter directly or indirectly the dormancy and germination of seeds. In this paper, inclusion of light as an additional factor to photoperiod and radiation in emergence models is explored and compared with the classical hydrothermal time (HTT) model using *Camelina microcarpa* as an example. HTT based on hourly estimates is also compared with that based on daily estimates. Results suggest that, although HTT based models are accurate enough for local applications, the precision of these models is improved when HTT is estimated hourly and solar radiation is included as a factor.

## Introduction

Knowledge about field emergence pattern of weeds and crops is important for better management and decisions in farming [1]. Therefore, it is important to develop dormancy release/induction and germination/emergence models for seeds as they could help predict critical periods for plant management [2]. However, difficulties arise when deciding threshold values for certain factors that affect these processes. Nevertheless, the effects of temperature, humidity, and light in the dormancy release/induction and germination processes have been studied thoroughly [3]. Many models have been developed for seedling emergence. Thermal time (TT) models often are appropriate for summer weeds, whereas hydrothermal time (HTT) models may be more suited for winter weeds [4]. These models usually are very accurate, especially if they are applied in the areas where the original experiments were performed. However, they sometimes fail to describe the emergence of certain species grown in other climatological areas. This was the case with *Thlaspi arvense* [5]. The authors studied the emergence of this weed over two years in three climatologically contrasting sites: Spain, Minnesota (USA), and Latvia. The resulting model based on HTT, developed with the Spanish data, failed when used with the data obtained in Minnesota and Latvia. The authors added daylight as a factor to correct the estimated HTT and created a new unit called photohydrothermal time (PhHTT). The resulting model substantially improved emergence predictions in the latter sites.

Including daylight in emergence models may seem unnecessary, as emergence itself does not need light, but germination is part of the emergence process [6]. Temperature fluctuation as well as exposure to light are known to affect dormancy and influence seed germination of many weed species [3; 7], such as *Polygonum aviculare* [8]. The effects of light on germination are also known to have important implications for emergence in the field [9]. Therefore, gaining a better understanding of the effect of photoperiod on weed emergence may help to improve the accuracy of emergence models. In this process, light could be incorporated as the amount of radiance received per square metre. In contrast to daylength, radiance is a measure of light quantity, which affects germination and other factors [10]. However, whether daylength and/or radiance can affect germination sufficiently to justify including them as factors to describe the process of emergence is unknown.

The objective of this work was to determine the best way of introducing light as a factor in the calculation of PhHTT models and to compare their accuracy with the classical HTT emergence models. As an example we used *Camelina microcarpa* grown in a Mediterranean climate (Lleida, Spain) and continental temperate climates (Morris, Minnesota, USA; and Riga, Latvia). Additionally, given the possibility of estimating hourly temperature and moisture conditions with the software developed by Spokas & Forcella [11], the developed models were compared to an hourly based HTT model.

## Materials and Methods

### General set up and site descriptions

Seeds of *Camelina microcarpa*, a threatened arable weed with potential as an oilseed crop, were harvested at maturity between June and July 2011 and 2012 in Camarillas (40°38’39”N-0°48”35”W, Teruel, Spain) and stored dry under laboratory conditions. Sets of seeds were sent to Morris and Riga. In Spain, the experiment was performed in a small part of a farm (with permission of the owner). Since this weed species is common in Spain, no additional permission was required. Also, in this commercial farm, no endangered species were present at the time the experiment was conducted. The second and third authors belong to the United States Department of Agriculture (USDA) and they obtained permissions for the experiment as well. This species is also present in North America and it is not an important weed in cropping systems. The experiment in Riga was performed in the Botanical Garden of the University of Latvia.

Trials were conducted from autumn to spring in two consecutive seasons (2011–12 and 2012–13) in fallow plots in Almenar, Spain (41°46’36”N-0°32’7”E) and in an experimental field in Morris, MN, USA (45°43’36”N-95°49’17”W). In Riga (56°57’02”N-24°06’57”E), seeds were sown in spring 2012. Viability of the seeds was tested by germination in growth chambers, and a tetrazolium test (incubation in 1% triphenyltetrazolium chloride solution in dark at 30°C for 24h) was conducted on non-germinating seeds. Initial viability of the seeds ranged between 82 and 92%.

Seeds were sown 1 cm deep in 1 m^2^ plots at a rate of 1000 seeds per plot, with four replications. *C*. *microcarpa* was one of six weed species that were studied in a randomized complete block experiment. The 1 cm depth was chosen to simulate cropping situation as recommended for *Camelina sativa* [12]. Sowing was done in four rows per plot, each with 250 seeds, to facilitate hand weeding of the plots and to simulate crop conditions. Organizing the seedlings in rows also reduced the potential for volunteer seedlings to enter into the study. Emergence of seedlings was followed weekly until May and newly emerged seedlings were identified with coloured wires. In Morris, seeds were sown on 19 and 18 September, in 2011 and 2012, respectively. In Almenar, sowing was delayed until 2 November in 2011 due to inclement weather, and was done on 4 October in 2012. In Riga seeds were sown on 1 May 2012. Plots were not watered, so that emergence of *C*. *microcarpa* was conditioned mainly by climatic conditions. All sites were free of *C*. *microcarpa* before sowing and there was no historical evidence of its presence.

### Weather data

Daily rainfall, maximum and minimum air temperatures, and solar radiance were obtained from a meteorological station situated 4 km away in Almenar, while in Morris they were obtained from a standard meteorological station located at the experimental field (http://www.ars.usda.gov/Services/docs.htm?docid=3512). The weather data set for Riga were obtained from a meteorological station at the University of Latvia (Latvian Environment, Geology and Meteorology Centre; http://www.meteo.lv/en/meteorologija-datu-meklesana/?nid=924). Daylight hours are those corresponding to each site and day, obtained from the agrometeorological services (www.ruralcat.cat; www.timebie.com).

### Model development

The models were developed with data from autumn-winter emergence periods of *C*. *microcarpa* grown in Almenar in seasons 2011–12 and 2012–13. Simulated soil temperatures (TT) and water potentials (hydrotime, HT) were used to calculate HTT based on the equation described by Roman *et al*. [13]:

HTT= ∑(HT×TT) (1)

where HT = 1 when *ψ* > *ψ*_*b*_, otherwise HT = 0; and TT = *T* − *T*_*b*_ when *T > T*_*b*_, otherwise TT = 0. *ψ* is the daily average water potential in the soil layer from 4 to 6 cm; *ψ*_*b*_ is the base water potential for seedling emergence; *T* is the daily average soil temperature in the soil at 1 cm depth and *T*_*b*_ is the base temperature for seedling emergence [14; 15]. With this formula, growing degree days are accumulated only when the *ψ* and *T* conditions were higher than *ψ*_*b*_ and *T*_*b*._ A soil depth of 1 cm depth was chosen for *T* because seeds were buried at that depth and temperature is the main factor affecting germination. Soil depth of 4–6 cm depth was chosen for *ψ* because seedlings must lengthen their radicles to a certain depth before emerging, so that they can absorb enough water to emerge and because this was the depth range that gave the best accuracy when fitting the HTT model.

The HTT was estimated using the Soil Temperature and Moisture Model (STM²) [11]. STM² requires, as input, daily maximum and minimum air temperatures and daily precipitation, including information on the geographical location and soil texture and organic matter. HTT were accumulated over days beginning on the sowing date. *ψ*_*b*_ and *T*_*b*_ were determined iteratively calculating HTT using a set of water potentials (-10.0 MPa to -0.5 MPa at -0.1 MPa intervals) and temperatures (-5° to +2°C at 0.1°C intervals). Namely, the scale of HTT was changed by modifying the *ψ*_*b*_ and the *T*_*b*_ until the highest accuracy (*R* ^*2*^) was obtained for the relationship between HTT and cumulative emergence of *C*. *microcarpa*.

To improve model accuracy and compensate for differences in emergence patterns at the three sites, estimated HTT was corrected by proportional daylight hours, considering daylight of 24h = 1, 12h = 0.5 and 0h = 0. The new unit is hereafter designated PhHTT, which is the product of HTT and day length. PhHTT was estimated in two different ways to assess the better method. In the first method, HTT was multiplied by its corresponding proportional daylight (PhHTT-1), and in the second method, the proportional daylight (PhHTT-2) was added to original HTT as follows:

$$\text{PhHTT}-1=\;\sum \left({\text{HTT}}_{t}\times D_{t}\right)$$ (2)

$$\text{PhHTT}-2=\;\sum \left[{\text{HTT}}_{t}+\left({\text{HTT}}_{t}\times D_{t}\right)\right]$$ (3)

Where HTT_*t*_ is the hydrothermal time in day *t*, and *D*_*t*_ is the proportional daylength in day *t*. Daily solar radiance (*SR*) was also included, creating the solar hydrothermal time (SHTT) and the combination of solar radiance with proportional daylength (PhSHTT) was also estimated as follows:

$$\text{SHTT}=\;\sum \left[\left({\text{HTT}}_{t}\times SR_{t})/(\text{ln}SR_{t}\times 100\right)\right]$$ (4)

$$\text{PhSHTT}-1=\;\sum \left({\text{SHTT}}_{t}\times D_{t}\right)$$ (5)

The process for developing PhHTT-1, PhHTT-2, SHTT and PhSHTT started with the parameters (*T*_*b*_, *ψ*_*b*_) estimated for the HTT based model. Our method of including photoperiod was different than that of Deen *et al*. [16], who used photothermal time to predict postemergence plant growth. As explained earlier, because germination is part of the emergence process, a logical extension is the use of photothermal time to calculate PhHTT to predict emergence.

The functional relationship between cumulative emergence and HTT, PhHTT-1, and PhHTT-2 was described by a four parameter Weibull model as follows:

y=a [1− e−(x−x50+ bln21cb)c] (6)

where *y* is 0 if:

x < x50−bln21c

*y* is the percentage of emergence, *x* is time expressed as HTT, and *a*, *b*, *c* and *x*_*50*_ are empirically derived constants: *a*, is the maximum percentage of emergence recorded, *b* is the rate of increase, *c* is a shape parameter and *x*_*50*_ is the HTT required to obtain 50% of maximum emergence. To make this Weibull model simpler, *a* was assumed to be 100% for each plot in each season. Fitting of the four parameter Weibull function for cumulative emergence was performed using SigmaPlot 11.0.

### Validation of the emergence model

The developed models were validated with emergence data from the different sites: first, autumn-winter emergence data of *C*. *microcarpa* sown in Morris in 2011–12 and 2012–13; and second, spring seedling emergence data from Riga in 2012. Agreement between predicted and actual emergence values was determined with the root-mean-square error predictor (RMSEP) as follows:

$$RMSEP=\sqrt{1/n\sum_{i=1}^{n}{\left(x_{i}-y_{i}\right)}^{2}}$$ (7)

where *x*_*i*_ represents actual cumulative percentage of emergence, *y*_*i*_ is predicted cumulative percentage of emergence, and *n* is the number of observations [17]. RMSE provides a measurement of the typical difference between predicted and actual values in units of percentage seedling emergence. The lowest RMSEP values indicated that the emergence model fit had been optimized.

### Emergence model based on hourly HTT

The STM² model developed by Spokas & Forcella [11] provides the option of estimating hourly HTT data. This allows the light factor to be considered as daylight hours when estimating the HTT, and the correction with daylength would not be needed. The accuracy of the model based on hourly data was compared with that from the daily HTT and PhHTT-1 and -2. Estimation of hourly HTT data was performed based on Eq 1 as follows:

$$\text{HTT}=\;\sum \left({\text{HT}}_{h}\times {\text{TT}}_{h}\right)/24$$ (8)

Where HT_*h*_ and TT_*h*_ are hydrotime and thermal time estimated as in Eq 1, but for every hour *h*. In order to avoid large values, cumulate HTT was divided by 24 (hours).

### Statistical analysis

Differences in total percentage emergence among locations (Almenar *vs* Morris *vs* Riga) and between seasons (2011–12 *vs* 2012–13) were analysed with two-way ANOVA. If interactions were found between factors, one-way ANOVA was performed to separate the respective factors. Significances were considered at P<0.05 level. To satisfy normality and homogeneity of variance, emergence percentages were transformed by the function arcsin(root(x/100)) if needed. Statistical analysis was performed with SPSS 15.

## Results

### Climatic characteristics of Almenar, Morris, and Riga

The three sites differed in temperature and precipitation (Table 1). The 2012–13 growing season was cooler than 2011–12 in both Almenar and Morris. Furthermore it was cooler in Morris than in Almenar. Also, 2011–12 was dryer in Almenar mainly during the winter period (Dec-March), while in Morris 2012–13 was very dry because of a spring drought (April-May). All the rainfall in autumn 2012–13 in Morris occurred after 17 October. In Riga, spring 2012 was characterized by cool and slightly humid weather (Table 1).

**Table 1:** Weather characteristics of Almenar, Morris and Riga in 2011–12 and 2012–13 during the periods when the experiment was carried out. Autumn: October-November; Winter: December-March; Spring: April-May.

		Mean Temperature (°C)			Precipitation (mm)		
Location	Season	Autumn*	Winter	Spring**	Autumn*	Winter	Spring**
Almenar	2011–12	13.5	6.1	14.4	101	3	122
	2012–13	12.2	6.2	12.3	122	51	145
Morris	2011–12	11.3	-4.9	12.2	41	9	166
	2012–13	10.2	-9.0	8.1	40	9	7
Riga	2012			14.0			115

*Sept-mid Nov in the case of Morris;

** May-June in the case of Riga.

### Emergence differences between locations

Although being Spanish in origin, *C*. *microcarpa* showed higher emergence percentages in Morris than in Almenar in both seasons (Table 2). Data from Riga were much more similar to Almenar than Morris.

**Table 2:** Total autumn and spring emergence (% of seed sown ± SE) in 2011–12 and 2012–13 for *Camelina microcarpa*, and total emergence in Riga in spring 2012. Aut-Wint columns refer to total emergence that occurred during autumn or autumn and winter. Different letters represent significant differences; lowercase letters: differences between localities and years in the same season (Aut-Wint or Spring); uppercase letters: differences between growing seasons within locality.

	Total emergence (% sown seeds)			
	2011–12		2012–13	
Location	Aut-Wint*	Spring	Aut-Wint	Spring
Almenar	2.6±0.3 bA	0.1±0.1 cB	2.8±0.4 bA	0.03±0.0 cB
Morris	9.5±2.3 aA	0.6±1.1 bB	11.1±2.3 aA	1.9±0.5 bB
Riga		4.7±1.1 a		

*Two way ANOVA indicated significant interaction between factors. Thus, factor accession was analysed for each location separately.

**Fig 1:**
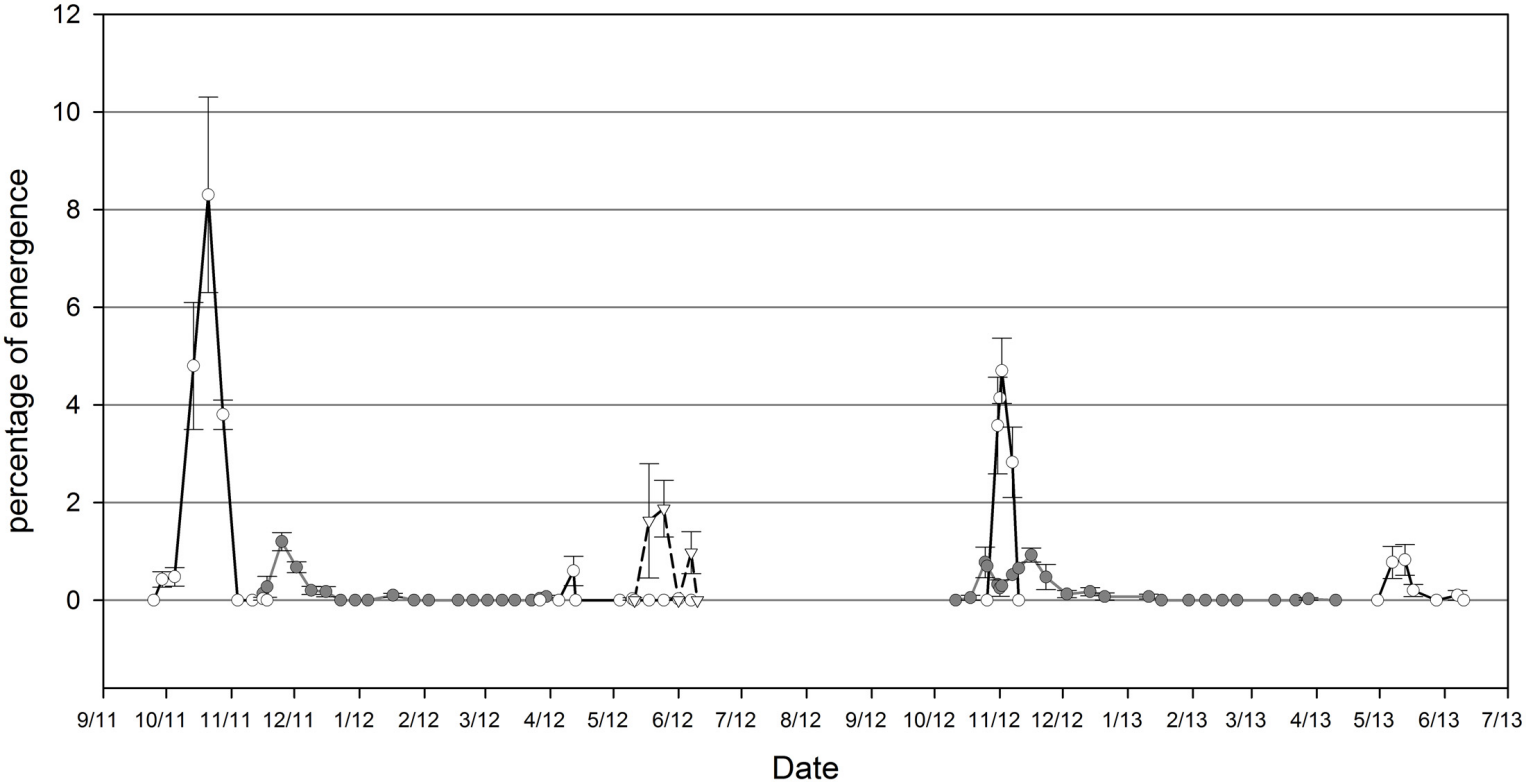
Distribution of the percentages of emergence of Table 1 throughout the two growing seasons, 2011–12 and 2012–13. (Grey line) Almenar. (Black line) Morris. Dates in the x-axis are in month/day/year.

In addition to differences in total emergence distributed throughout the seasons, emergence was delayed in Morris during autumn 2012 compared to Almenar (Fig 1). This delay was caused by the drought conditions in Morris in autumn 2012 (Table 1).

### Emergence models development

**Fig 2:**
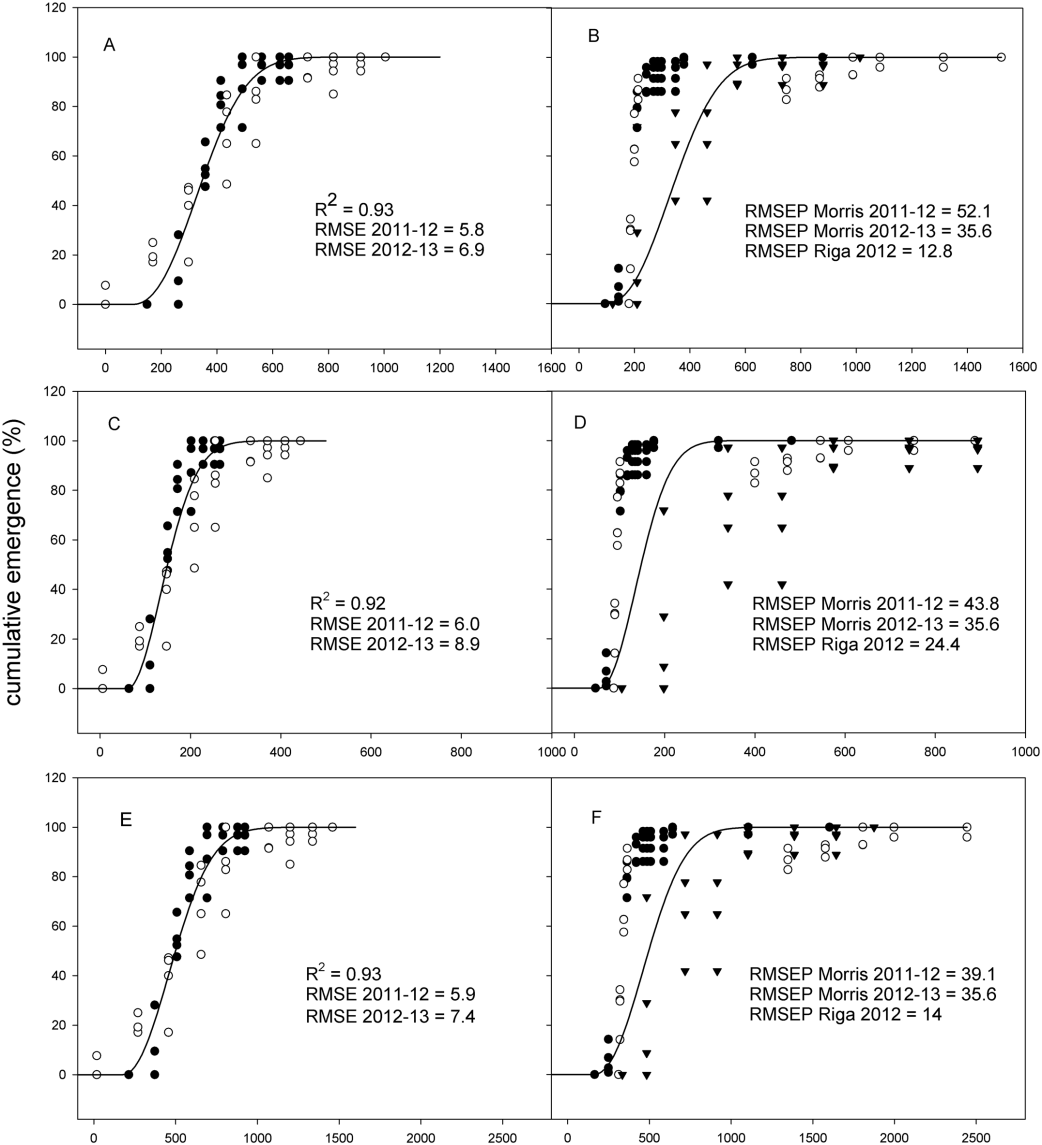
Development and validation of models based on HTT, PhHTT-1 and PhHHT-2. Left, development of the (A) HTT, (C) PhHTT-1 and (E) PhHTT-2 based emergence models with data from Almenar (Spain) 2011–12 (●) and 2012–13 (○), with the corresponding *R* ^*2*^ and the RMSE for each series of data; Right, validation of the models (B, D and F) developed in Almenar with data sets from Morris in 2011–12 (●) and 2012–13 (○), and Riga (▼), and their corresponding RMSEP. HTT, hydrothermal time; PhHTT-1, photohydrothermal time estimated with the corresponding proportional daylight; PhHTT-2, photohydrothermal time estimated with the corresponding proportional daylight plus the original HTT. *R* ^*2*^ is the coefficient of determination; RMSE is the root mean square error; RMSEP is the root mean square error for prediction.

**Fig 3:**
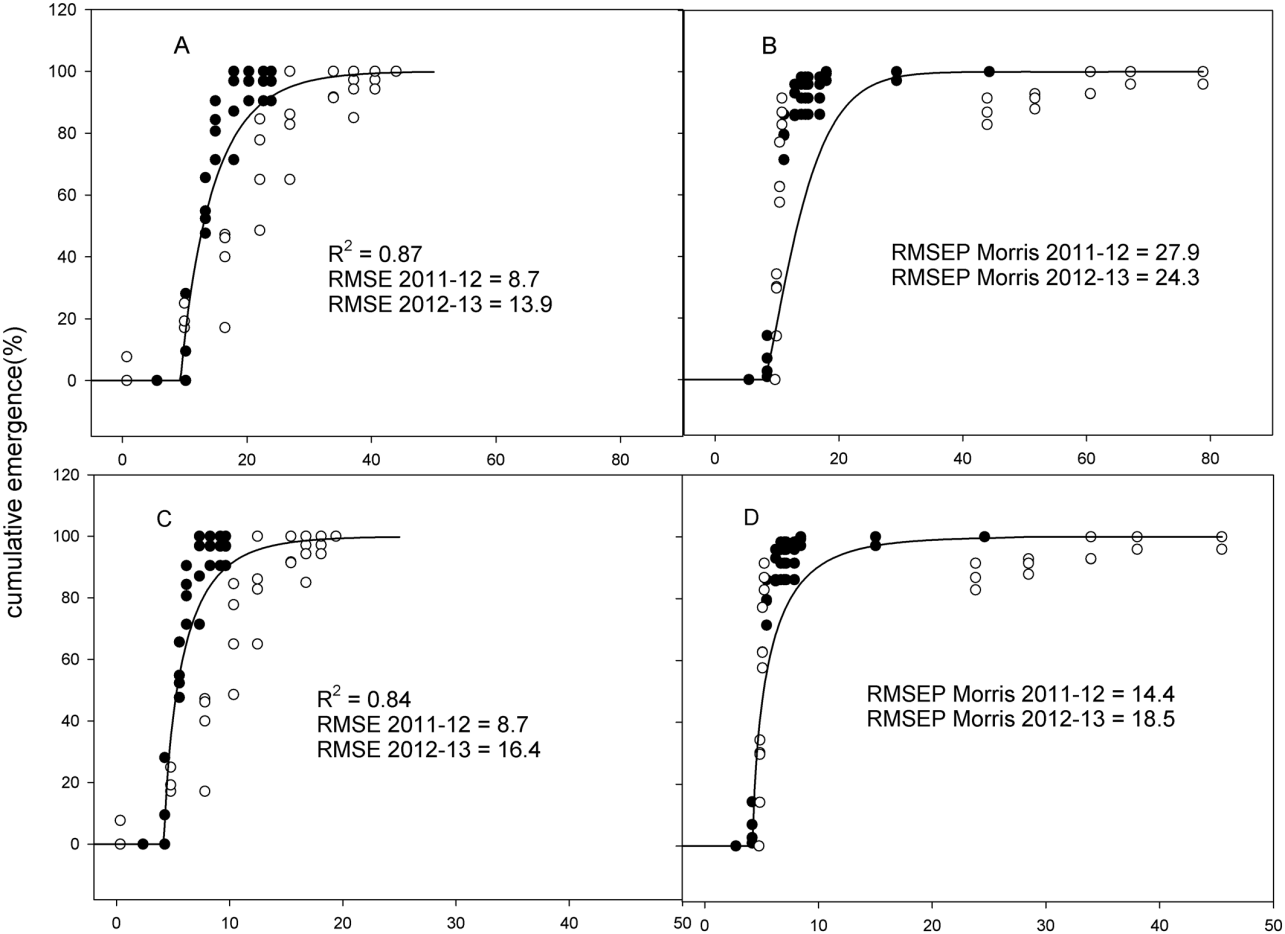
Development and validation of models based on SHTT and PhSHTT. Left, development of the (A) SHTT and (C) PhSHTT based emergence models with data from Almenar (Spain) 2011–12 (●) and 2012–13 (○), with the corresponding *R* ^*2*^ and the RMSE for each series of data; Right, validation of the models (B and D) developed in Almenar with data sets from Morris in 2011–12 (●) and 2012–13 (○), and their corresponding RMSEP. SHTT, solar hydrothermal time; PhSHTT, photosolar hydrothermal time, estimated with SHTT and the corresponding proportional daylight (for more details see Material and methods). *R* ^*2*^ is the coefficient of determination; RMSE is the root mean square error; RMSEP is the root mean square error for prediction.

*C*. *microcarpa* tended to emerge mainly in autumn-winter, with one important peak between October and December, followed by a small spring peak, which was almost negligible in Almenar (<1%) and more abundant in Morris (between 2 and 16%) (Fig 1). The initiation of HTT accumulation was established at the time of sowing. *T*_*b*_ was estimated at -3.1°C, and *ψ*_*b*_ at -3.8 MPa. Estimates of the variables *b*, *c* and *x*_*0*_ fitted to HTT, PhHTT-1, PhHTT-2, SHTT and PhSHTT for *C*. *microcarpa* are presented in Table 3, and RMSEP for each series of validation data (Morris and Riga) are shown in Figs 2 and 3.

**Table 3:** Estimates of the variables *b*, *c* and *x*_*0*_ fitted to HTT, PhHTT-1, PhHTT-2, SHTT, PhSHTT and hourly HTT, as well as their respective *R* ^*2*^, *F* value and significance. *b* is the rate of increase, *c* is a shape parameter and *x*_*50*_ is the HTT required to obtain 50% of maximum emergence. HTT, hydrothermal time; PhHTT-1, photohydrothermal time estimated with the corresponding proportional daylight; PhHTT-2, photohydrothermal time estimated with the corresponding proportional daylight plus the original HTT; SHTT, solar hydrothermal time; PhSHTT, photosolar hydrothermal time, estimated with SHTT and the corresponding proportional daylight (for more details see Material and methods); *R* ^*2*^ is the coefficient of determination, *F* is the value of Fisher’s F distribution; *P* is the significance of the model adjustment.

	*b*	*c*	*x*_*0*_	*R* ^*2*^	*F*	*P*
HTT	297.4	2.337	346.7	0.93	335.1	<0.0001
PhHTT-1	111.7	2.079	148.2	0.92	276.3	<0.0001
PhHTT-2	405.0	2.235	495.4	0.93	322.9	<0.0001
SHTT	7.1	1.271	13.3	0.87	159.3	<0.0001
PhSHTT	1.6	0.669	5.2	0.84	124.7	<0.0001
Hourly HTT	340.3	2.289	384.5	0.93	356.2	<0.0001

Results indicated that HTT could be considered as the best method of developing an emergence model. The highest *R* ^*2*^ and lowest RMSE estimated for the data sets used for the model development were obtained with the classical method of estimating HTT, closely followed by PhHTT-2, PhHTT-1, SHTT and PhSHTT (Figs 2 and 3). However, the application of the HTT based model to data from the other sites failed to describe the emergence in Morris accurately, but the prediction was better in Riga (Fig 2B). The approximation to describing emergence was progressively improved with PhHTT-1, PhHTT-2 and SHTT, and the lowest RMSEP was achieved with PhSHTT, with values below 20 in every case (Figs 2 and 3). The lack of information of solar radiation in Riga prevented us from estimating the last two approximations with SHTT and PhSHTT, and the best RMSEP for Riga was obtained with PhHTT-2 (Fig 2F).

**Fig 4:**
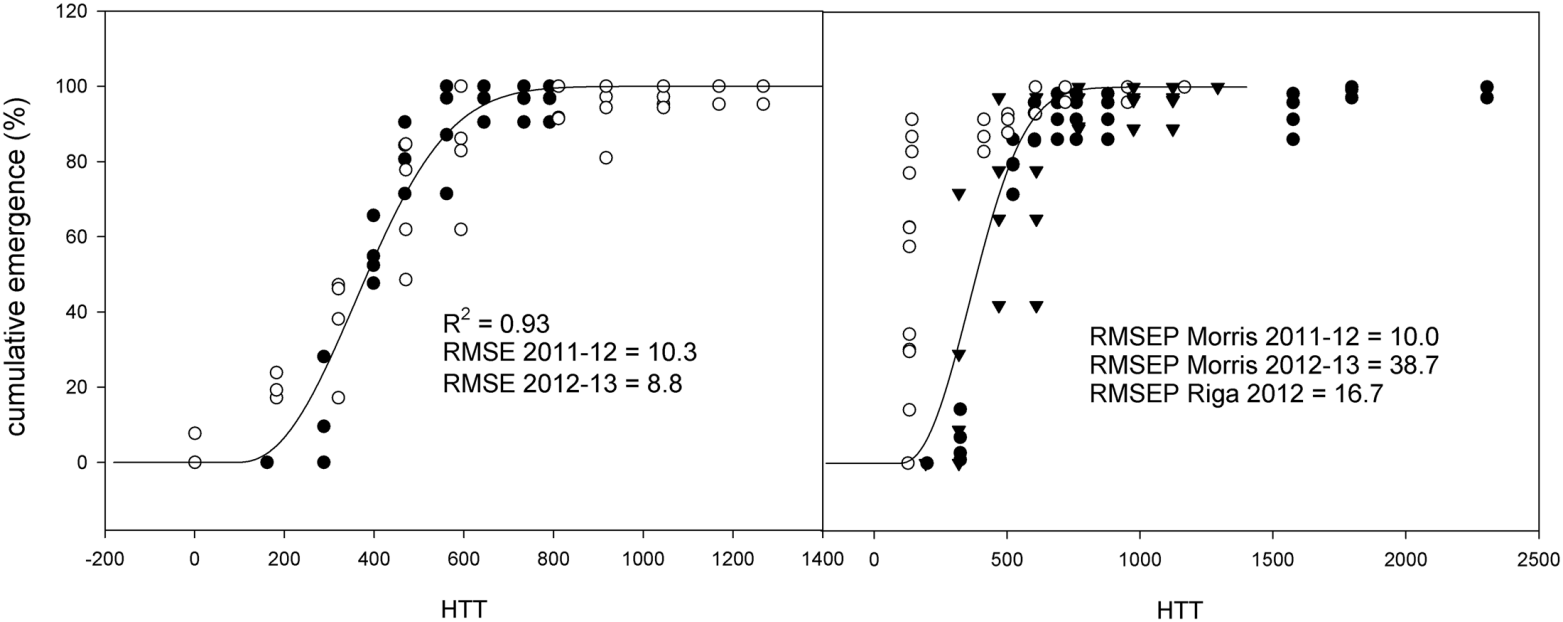
Development and validation of the hourly based HTT model. Left, development of the hourly HTT based emergence model with data from Almenar (Spain) 2011–12 (●) and 2012–13 (○), with the corresponding *R* ^*2*^ and the RMSE for each series of data; Right, validation of the model developed in Almenar with data sets from Morris in 2011–12 (●) and 2012–13 (○), and Riga (▼), and their corresponding RMSEP. *R* ^*2*^ is the coefficient of determination; RMSE is the root mean square error; RMSEP is the root mean square error for prediction.

The results obtained with the alternative hourly HTT model were similar to those obtained by either HTT, PhHTT-1 or PhHTT-2, but RMSE was improved, resulting in values below or near 10 (Fig 4). With respect to the application of the hourly HTT model to other sites, it improved the description in Morris in 2011–12 and in Riga, but failed to describe emergence in Morris in 2012–13.

## Discussion

Since the seeds were sown earlier in Morris, the emergence period also started earlier in the season than in Almenar. However, the emergence period lasted longer in Almenar due to milder autumn and winter conditions. All models were able to describe the emergence in Riga, with the HTT and PhHTT-2 based models providing good fits with RSMEP values < 20.

*C*. *microcarpa* is an autumn emerging arable species in Spain, whereas this characteristic is occasional in temperate climates like Canada [18] and Minnesota. Its emergence period spanned two seasons from mid-October or early November to early January, in Almenar. In contrast, >84% of the emergences occurred before mid-November in Morris, with only a small spring flush in April-May. The rapid decrease in temperature that occurred in November in Morris would have prevented some seeds from germinating, and these late germinating seeds would have appeared as seedlings in spring, like in *Thlaspi arvense* [5].

### Models development and validation

Despite the differences observed in the emergence timing at the three sites during the two growing seasons, the use of HTT was able to equalize the differences between seasons in each site. The inclusion of daylength was demonstrated recently to improve emergence models [5], and when HTT was combined with solar radiation and daylight hours (PhSHTT), the resulting model integrated the differences among sites. This was achieved by considering parameters at the exact soil depth where the seeds and the seedlings were affected.

Contrary to previous works, temperature for the HTT accumulation was considered only at 1 cm because this was the depth at which seeds were buried, while moisture was considered between 4 and 6 cm. In the emergence process, temperature affects germination (and dormancy release) of the seeds, while moisture is a key factor for radicle and hypocotyl elongation, which ultimately allows emergence (i.e., the moment seedlings break the soil surface). Therefore, despite the lack of moisture in the superficial soil layers, seedlings can emerge if the moisture in the 4–6 cm soil layer is adequate to maintain turgor and hence, plant tissue growth.

In the present study, soil temperature was estimated at 1 cm because of the placement of the seeds. However, the seed distribution of natural populations often varies within the soil profile. Therefore, temperature should be considered for the entire profile from where most seedlings emerge but this can differ for particular weed species. For instance, this depth will most likely be the upper 2 cm of the soil for most small-seeded species [19], but could extend to below 7 cm for large-seeded species, such as *Avena fatua* [20].

The use of hourly estimated HTT improved the prediction of emergence compared to daily HTT, PhHTT-1 and PhHTT-2, suggesting that this method integrates the corrections and improvements obtained with the inclusion of daylight hours in the daily based models. These results are consistent as hourly estimation of HTT uses 48 temperature data points (hourly maximums and minimums), compared to the two data points (daily maximum and minimum temperatures) used for the daily estimation of HTT. However, hourly data may not be readily available.

### Implications for emergence model development

Although some authors distinguish different processes within the emergence phenomenon (dormancy release, germination and early preemergence growth) and develop submodels for each [2; 21], the aim of this work was to try to develop a single model that could describe the emergence particularly for *C*. *microcarpa*, irrespective of seedling density. These two strategies of modelling emergence are not contrary, but could be complementary, as our model that includes daylength could also be considered as a submodel for the original HTT based model.

HTT based models (whether daily or hourly) have been demonstrated to describe accurately the emergence of weeds. However, they often are limited to specific or local climatic areas (e.g., Mediterranean climate or northeastern Spain). The fact that these models were the most accurate, with RMSE values near or below 10%, and not improved with the inclusion of the daylength factor, confirms this theory. For hourly HTT, daily variation of HTT hour by hour includes the daylength factor effects on soil temperature and moisture, but not its direct effect on the seeds, for which, solar radiation is a better parameter to be included.

Thus, HTT models could be an easy tool for predicting weed emergence for local weed management decisions. This implies, however, the development of a different model for each climatic or geographic region, as demonstrated for the emergence data in Morris, and the need for sampling the emergence in each of these sites.

Contrary to our results for *C*. *microcarpa*, Royo-Esnal *et al*. [5] demonstrated that including daylight (as PhHTT-1) in a predictive model significantly improved the accuracy of the model developed for *Thlaspi arvense* emergence at the original site, and to a lesser degree, its accuracy for predicting emergence in other climatic regions. These differences in the model improvement from one species to another suggest a variation of the light effect among species.

Although accuracy of the prediction could decrease locally, as in this work, including light as a factor permits more accurate descriptions of the emergence in other climatic and geographic areas, and it represents a step forward towards developing a unique worldwide model for a certain species. The techniques for including light as a factor that have been presented here are merely examples of how it might be accomplished. Many improvements may still be needed. Regardless, the aim of this work was to demonstrate the need for inclusion of light as a component developing a common model for a specific weed species that could be applied universally.

## Conclusions

The inclusion of light to develop emergence models is an important step forward in understanding the seedling emergence process. The use of PhSHTT could permit the development of common models for several sites, thereby saving time and effort. Better methods of including solar radiance and/or daylength are likely, as are additional factors that have not been considered in this work. Properly selecting the soil layers for temperature and moisture recordings is also important for obtaining accurate models. HTT models could be more accurate locally, while PhSHTT-based models seem more reliable for predicting emergence across sites with large climatological contrasts (e.g., southern Europe vs. northern USA). Lastly, the use of hourly estimated HTT consistently improved the prediction of emergence compared to the daily based HTT estimation.
